# Operational Deflection Shape Measurements on Bladed Disks with Continuous Scanning Laser Doppler Vibrometry

**DOI:** 10.3390/s24113413

**Published:** 2024-05-25

**Authors:** Cuihong Liu, Tengzhou Xu, Tao Chen, Shi Su, Jie Huang, Yijin Li

**Affiliations:** 1 School of Aeronautic Engineering, Nanjing Vocational University of Industry Technology, Nanjing 210023, China; 2018100938@niit.edu.cn (T.X.); taochen@niit.edu.cn (T.C.); shi.su@niit.edu.cn (S.S.); 2004100333@niit.edu.cn (J.H.); 2021101199@niit.edu.cn (Y.L.); 2 Aeronautic Intelligent Manufacturing and Digital Health Management Technology Engineering Research Center of Jiangsu Province, Nanjing 210023, China

**Keywords:** bladed disk, laser Doppler vibrometry, ODS FRF, phase identification

## Abstract

The continuous scanning laser Doppler vibrometry (CSLDV) technique is usually used to evaluate the vibration operational deflection shapes (ODSs) of structures with continuous surfaces. In this paper, an extended CSLDV is demonstrated to measure the non-continuous surface of the bladed disk and to obtain the ODS efficiently. For a bladed disk, the blades are uniformly distributed on a given disk. Although the ODS of each blade can be derived from its response data along the scanning path with CSLDV, the relative vibration direction between different blades cannot be determined from those data. Therefore, it is difficult to reconstruct the complete vibration mode of the whole blade disk. In order to measure the complete ODS of the bladed disk, a method based on ODS frequency response functions (ODS FRFs) has been proposed. While the ODS of each blade is measured by designing the suitable scanning paths in CSLDV, an additional response signal is obtained at a fixed point as the reference signal to identify the relative vibration phase between the blade and the blade of the bladed disk. Finally, a measurement is performed with a simple bladed disk and the results demonstrate the feasibility and effectiveness of the proposed extended CSLDV method.

## 1. Instruction

A bladed disk is one of the important components of rotating machinery such as aeroengines, turbines and impellers, etc. High vibratory stresses of turbomachinery components can be a major cause of vibration cracks and even breaking for blades [1,2,3], so the dynamic behavior of bladed disks has always interested the engineering community over the years. In recent decades, there have been several numerical methods used to predict the dynamic characteristics of bladed disks via numerical modeling [3,4] and finite element modeling (FEM) [5,6]. These studies indicate that the bladed disk is highly sensitive to mistuning, such that even small deviations in the structural properties of individual sectors of the bladed disks can result in the localization of vibration energy and a significant increase in forced responses. There are many causes that can change the properties of the nominal design. However, it is very difficult to take uncertain factors into account when establishing finite element modeling.

To more precisely explore the dynamic features of the bladed disk structure, an experimental study is indispensable. Zhao Z B et al. [7] took the bladed disk with 12 blades as an example to conduct an experimental study on the natural characteristics of the tuned and detuned bladed disks. Yao J et al. [8,9] studied the spatial distributions of the mistuned mode shapes and the mode location of a simplified bladed disk, demonstrating the deformation characteristics of all the modes within the experimental frequency band and the deformation differences among the various modes through the test data of 120 measuring points. To experimentally acquire the modal shape of the bladed disk, a large amount of measuring points need to be arranged on the surface of the bladed disk. However, this greatly increases the time and cost of experimental testing and it is difficult to obtain accurate vibration mode shapes. Therefore, the experimental measurement of the operational deflection shapes of bladed disks is still a relatively demanding task.

In recent years, laser Doppler vibrometry (LDV), as a new non-contact measurement method, has been widely used in aerospace [10], automobile [11] and other fields due to its advantages of long-distance measurement, high measurement accuracy and no installation. Heinemann [12] et al. measured the natural frequencies and the modal shapes of a five-blade axial-flow fan with scanning laser Doppler vibrometry (SLDV). In fact, the density of measurement points has been improved to some extent, but also greatly increased the time-consuming nature and the storage requirements of test data. In order to further improve the test efficiency and the density of test points, continuous scanning laser Doppler vibrometry (CSLDV) is proposed based on SLDV, where the laser point, instead of dwelling at a fixed location, is continuously moving across a measurement surface along the designed path. The SLDV was used in vibrational measurements to extract only a few dozen measured points across the grid. However, the CSLDV is able to measure thousands of vibration points in a few seconds and reconstruct a more accurate ODS through the demodulation method, arranging each single ODS according to the position of scan paths on the measured structure. Therefore, this CSLDV is also called the full-field measurement technique. Sriram P. et al. [13] first applied the CSLDV to measure the vibration of a cantilever beam in the line scanning mode. With the application of the CSLDV, Y Hu et al. performed an experiment on a free-hanging aluminum plate in a thermal environment, and the findings revealed that the accuracy of the ODS was improved by 20 times compared to a manual-moving LDV method [14]. Additionally, there are also some studies reported on excitation methods for the CLSDV [15,16,17,18]. K Yuan et al. [19,20] obtained the modal parameters of a model turbine blade with a curved surface excited by white noise by the CSLDV, and the results demonstrated that the identified modal parameters possess significantly higher accuracy and efficiency in comparison to those from the SLDV.

Most of the studies reported on CSLDV techniques are mainly based on the simple continuous surface of structures, such as beams, plates, disks or a single blade [21], and the measurement can be completed at once along a continuous scanning path. However, there is still a great challenge for the testing of discontinuous multi surfaces of structures such as bladed disks. For the bladed disk, if the laser beam conducts a continuous scanning test within a large area covering the bladed disk, laser leakage will occur in the area without the bladed disk. And then in the output data of the LDV corresponding to the scanning trajectory, there will be some small segments of invalid data. The invalid data segments will reduce the accuracy of the reconstructed ODS. Therefore, it is best that the scanning path precisely matches the bladed disk. The continuous scanning path of the laser precisely switches from one blade to another to exactly cover the structure surface while also ensuring that the scanning frequency does not change, which is rather complicated. Because reconstruction of the ODS from the output data in CSLDV demanded that the scanning frequencies of the laser beam in the x direction and the y direction should be unchanged during the entire scanning process, this will pose great difficulties for the CSLDV applied to the bladed disk. 

To address these issues of the ODS testing of the bladed disk in CSLDV, this paper has also conducted some studies. The aim of the present paper is to extend the CSLDV techniques for bladed disk structures, in that a suitable continuous scanning path for a multi-blade disk structure is designed and the ODSs of all the blades can be derived from the sideband patterns of the CSLDV output spectrum. Then, a vibration phase identification method of all blades is needed to reconstruct the ODS of the whole-bladed disk. As an example, a 16-blade disk for vibration measurement is taken. A continuous scanning path suitable for full field testing in CSLDV is designed and the ODS of the bladed disk is reconstructed by identifying the vibration phase between each blade. The results show that the design of the continuous scanning path is feasible and the effectiveness of the identification method of the vibration phase in serving CSLDV is evaluated. 

The rest of this paper is organized as follows. Section 2 describes the theoretical basis of ODS reconstruction in CSLDV. In Section 3, the design of the measurement campaigns on a 16-blade disk is discussed, including how to switch the laser beam to scan the next test blade. The ODSs of all blades were reconstructed from the measured sideband spectrums. The identification method of the vibration phase between each blade is described in Section 4. The main purpose of this section is to obtain the relative vibration phase of all the blades and to reconstruct the ODS of the whole-bladed disk.

## 2. Theoretical Basis of ODS Reconstruction in CSLDV

**Figure 1:**
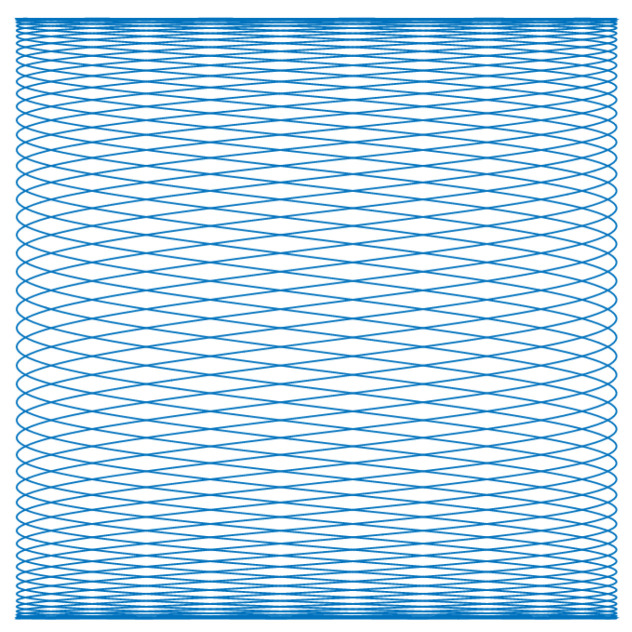
Continuous surface scanning path diagram.

The small damping structure will resonate when the excited frequency is near the natural frequency. The vibration response of the structure measured by the laser Doppler vibrometers, which can be expressed as follows:

(1) $$v(x,y,t)=d^{\prime }(x,y,t)=V_{R}(x,y)\cos\omega t+V_{I}(x,y)\sin\omega t$$

where v(x,y,t) is the vibrational velocity of point (x,y) at time *t*. $V_{R}(x,y)$ and $V_{I}(x,y)$ are the real and imaginary components of the vibration, respectively. They can be fitted by the polynomial series as follows:

(2) $$\begin{array}{l} V_{R}(x,y)=\sum_{m,n=0}^{p,q}V_{Rm,n}x^{m}y^{n}\\ V_{I}(x,y)=\sum_{m,n=0}^{p,q}V_{Im,n}x^{m}y^{n} \end{array}$$

where *p* and *q* are the polynomial order in the *x* and *y* direction, respectively. For a rectangular plate, the scanning path of the area sine scan moved on the surface of the structure can be expressed by two sinewaves equations when the scanning speed of the laser spot has a sine variation.

(3) $$\begin{array}{l} x=\cos(\omega_{sx}t)\\ y=\cos(\omega_{sy}t) \end{array}$$

where $\omega_{sx}$ and $\omega_{sy}$ are the scanning frequencies of the laser point along the X and Y directions, respectively. The scanning paths covered on the rectangular plates can be generated by dissimilar frequency sinusoids using sinusoidal trajectories, e.g., 2D Lissajou trajectories, as shown in Figure 1.

Substituting Equations (2) and (3) into Equation (1), and expanding out trigonometrically, the following form of the vibration signal is derived:

(4) $$v(x,y,t)=\sum_{m,n=0}^{p,q}A_{Rm,n}\cos[(\omega \pm m\omega_{sx}\pm n\omega_{sy})t]+\sum_{m,n=0}^{p,q}A_{Im,n}\sin[(\omega \pm m\omega_{sx}\pm n\omega_{sy})t]$$

where $A_{Rm,n}$ and $A_{Im,n}$ are the sideband spectrum amplitudes of real and imaginary components at different frequencies, respectively. According to Equation (4), the sideband spectrum of vibration velocity signal obtained by laser continuous surface scanning test consists of all components when *m* and *n* are taken as different values. It can be deduced that the transformation to recover the polynomial coefficients from the spectral amplitudes of the LDV output is:

(5) $$\begin{array}{l} {\left[A_{R}\right]}_{m\times n}={\left[T\right]}_{m\times m}{\left[V_{R}\right]}_{m\times n}{\left[T\right]}_{n\times n}^{T}\\ {\left[A_{I}\right]}_{m\times n}={\left[T\right]}_{m\times m}{\left[V_{I}\right]}_{m\times n}{\left[T\right]}_{n\times n}^{T} \end{array}$$

Each element of matrix *T* satisfies the following equation:

(6) $$T(i,j)=\left\{ \begin{array}{l} 0\;\;\;\;\;\;\;\;\;\;\;\;i+j\;\mathrm{is}\;\mathrm{odd},\;\;i>j\\ \frac{C_{i}^{i-j}}{2^{i}}\;\;\;\;\;\;\;i+j\;\mathrm{is}\;\mathrm{even} \end{array}\right.\;\;\;,\;\;\;i\;\mathrm{and}\;j\;\mathrm{are}\;\mathrm{natural}\;\mathrm{number}$$

By expanding the Fourier series of the vibration signal obtained from the output of CSLDV, the amplitudes of the sideband spectrum $A_{Rm,n}$, $A_{Im,n}$ and the transformation matrix *T* can be deduced. Therefore, the real and imaginary components of the operational deflection shape can be obtained as follows:

(7) $$\begin{array}{l} {}[V_{R}]={\left[T\right]}^{-1}[A_{R}]{\left[T\right]}^{T}\\ {}[V_{I}]={\left[T\right]}^{-1}[A_{I}]{\left[T\right]}^{T} \end{array}$$

## 3. ODS of Bladed Disc in CSLDV

### 3.1. Modal Characteristics of Bladed Disk

**Figure 2:**
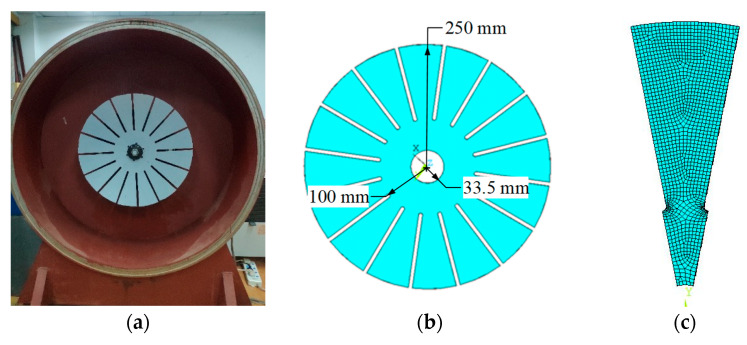
Bladed disk. (**a**) Bladed disk. (**b**) Model. (**c**) FEM of a single sector.

A bladed disk, as shown in Figure 2a, is a circularly symmetric structure whose modes are described by the mode shapes of a single sector and numbers of nodal diameter. The thickness of the structure is 2 mm, and the other dimensions are shown in Figure 2b. The finite element model (FEM) of a single sector is built with hexahedral elements, as shown in Figure 2c. Material properties are given as follows: the density and the Young’s modulus are 7840 Kg/m^3^ and 206 GPa, respectively. Poisson’s ratio is 0.3. 

**Figure 3:**
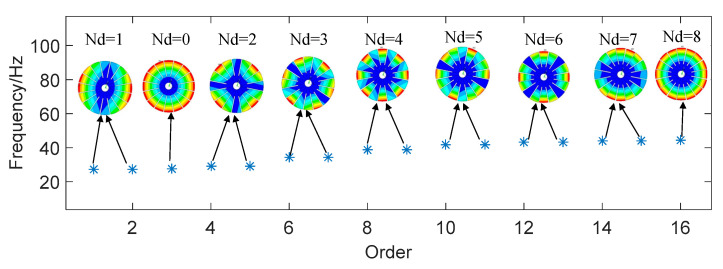
Modal shapes of different nodal diameters.

The modal frequencies of the first 16 modes derived from the FEM as listed in Table 1. The modal frequencies appear in pairs due to the characteristic of circularly symmetric structure, except for the modes which Nd = 0 and Nd = 8 for the 16-blade disk. The two modes of a modal pair have the same frequencies and orthogonal mode shapes, which are demonstrated in Figure 3 (only one of the mode shapes is shown for modal pairs). In the figure, the ‘*’ indicates the frequency values of different nodal diameters, and the arrow points to is the modal vibration mode corresponding to this frequency.

### 3.2. Measurement in CSLDV

#### 3.2.1. Design of the Continuous Scanning Path

The bladed disk shown in Figure 2a is employed to measure operational deflection shapes in CSLDV. The fan-shaped blades cannot be completely covered by the continuous scanning path of the laser spot described in Equation (3), so it is necessary to design a suitable continuous scanning path for the specific surface.

The scan pattern of the fan-shaped blades is much more complicated than the rectangular plate, as shown by the following:

(8) $$\begin{array}{l} x(t)=\left[\frac{R_{o}-R_{i}}{2}\cos(2\pi \omega_{sy}t)+\frac{R_{o}+R_{i}}{2}\right]\cos(\phi (t)+\tau )\\ y(t)=\left[\frac{R_{o}-R_{i}}{2}\cos(2\pi \omega_{sy}t)+\frac{R_{o}+R_{i}}{2}\right]\sin(\phi (t)+\tau ) \end{array}$$

where *R_o_* and *R_i_* are the initial radius and final radius of the scanning range in the radial direction, respectively. τ is the initial phase angular position, while ϕ(t) is the circumferential scanning control parameters which is described by the equation:

(9) $$\phi (t)=\theta \sin(2\pi \omega_{sx}t)$$

where θ is the edge amplitude of the blades.

**Figure 4:**
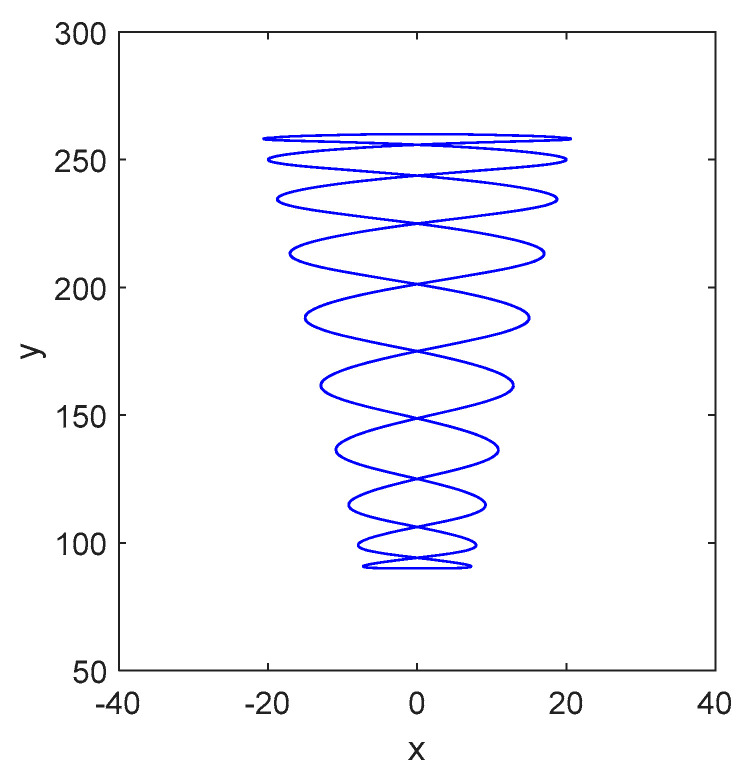
Scanning path on blades.

Take the scanning frequency $\omega_{sy}=3\;\mathrm{Hz}$, $\omega_{sx}=30\;\mathrm{Hz}$ as an example, the scanning path of the laser spot on the blade surface is shown in Figure 4.

For the different blades, the scanning pattern is the same except for the initial phase angular position τ. The blades are evenly distributed on the circumference of the disk; therefore, the initial phase angular position of each blade in Equation (8) is instead given by $\tau_{n}$, which can be expressed as:

(10) $$\tau_{n}=\frac{\pi }{2}+\frac{n-1}{N}\cdot 2\pi$$

where *n* is the number of blades, *N* is the total number of blades of the bladed disk. Therefore, the continuous scanning test of all blades could be carried out by changing different blade numbers *n* ( n=1,2,3,⋯,N). 

#### 3.2.2. Experimental Test

The scanning frequencies are set to $\omega_{sy}=1.1\;\mathrm{Hz}$ and $\omega_{sx}=10\;\mathrm{Hz}$, respectively. The sampling frequency *Fs* is 8192 Hz, which needs to satisfy Nyquist’s sampling theorem. In this experiment, the design of CSLDV is used to measure the fan-shaped blade, the vibration data is collected for 5 s, and then the next blade is switched for testing until all the blades are traversed. The experimental four-nodal diameter bending mode of the bladed disk measured by LDV is 36.88 Hz, and there is a deviation of 3.96% compared with the 38.40 Hz derived from the FEM. Thus, a sinusoidal signal with a single frequency ω=36.88 Hz was supplied to the electromagnetic exciter to excite the bladed disk in the test. It is known in advance that when the excitation frequency approaches or equals the natural frequency, the mode corresponding to that natural frequency can be well excited.

**Figure 5:**
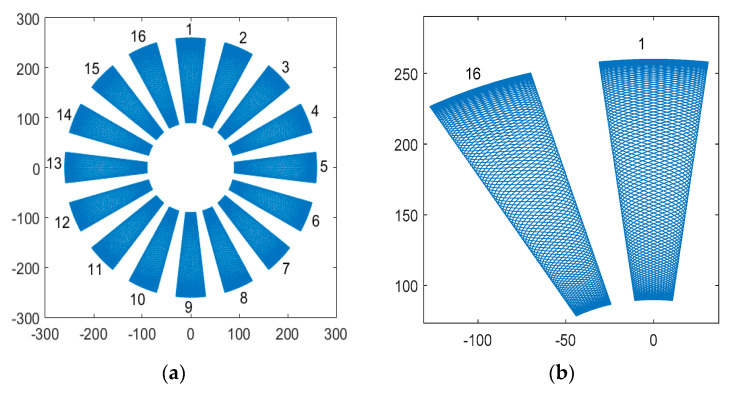
Continuous scanning path of all blades. (**a**) Scanning path. (**b**) Local zoom.

The continuous scanning path of all blades (No. 1–16) is shown in Figure 5.

**Figure 6:**
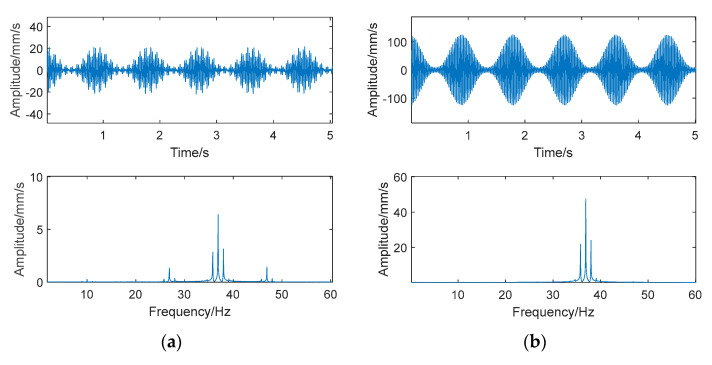

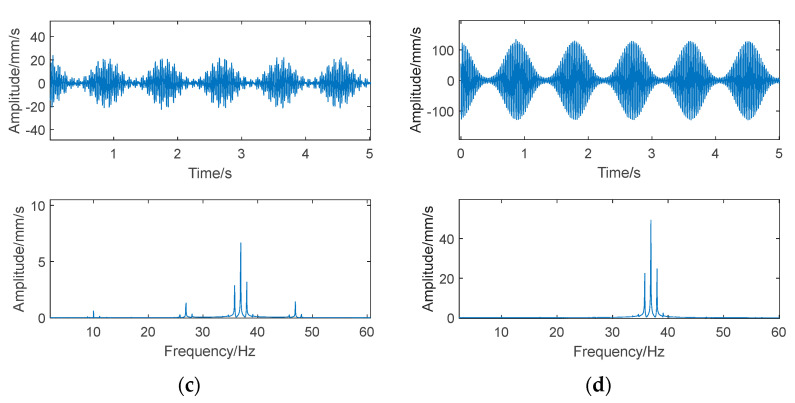
Vibration signals and sideband spectrums of blades. (**a**) No. 1. (**b**) No. 2. (**c**) No. 3. (**d**) No. 4.

The CSLDV measurements are carried out on all blades and the vibration signals in the time domain are obtained from the output of a laser Doppler vibrometer. Taking the first four blades as an example, the vibration signal and the sideband spectrums are shown in Figure 6. It can be found that the vibration forms of the odd-numbered blades shown in Figure 6a,c are consistent and their vibration amplitudes are relatively small, while the vibration forms of even-numbered blades shown in Figure 6b,d have the same form and larger amplitude. This phenomenon coincides with the four-nodal diameter mode of the first family mode of the bladed disk. The values of *m* and *n* are determined by the sideband spectrums. Specifically, according to the sideband spectrums shown in Figure 6, the values of the sideband number *m* and *n* of the odd-numbered blades are both 0 and ±1, while the values of the even blades are *m* = 0, *n* = 0, ±1, respectively.

The operational deflection shapes of all the blades have been reconstructed from the measured sideband spectrums, and are listed in Table 2. The results show that the vibration amplitude of the even-numbered blades is much larger than that of the odd-numbered blades. According to the number sequence and vibration amplitude of blades, it can be concluded that all the odd blades in the operation condition are located in the four-nodal diameter positions of the bladed disk.

Although all ODSs of the blades are obtained by using the extended CSLDV, the ODS of the whole-bladed disk cannot be directly reconstructed because of the unknown vibration phase of different blades, which cannot be derived from the sideband spectra of CSLDV. Therefore, it is necessary to identify the vibration phase between the blades for reconstructing the ODS of the whole-bladed disk.

### 3.3. Identification of Vibration Phase and ODS Reconstruction of the Whole-Bladed Disk

**Figure 7:**
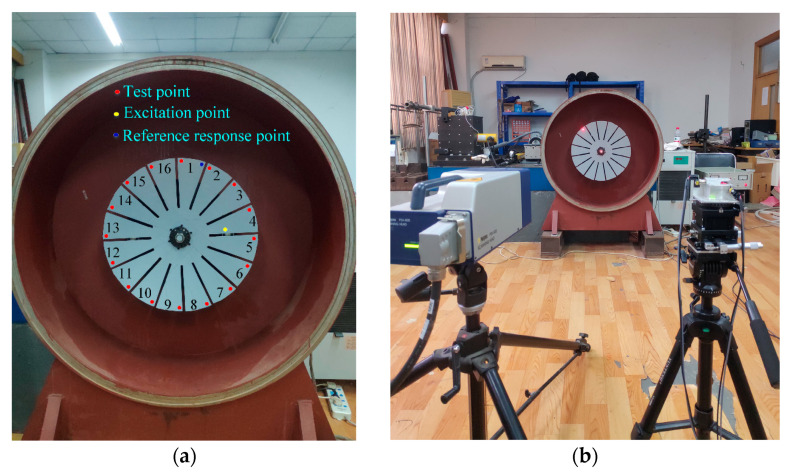
Testing scheme. (**a**) Location of measuring points. (**b**) Measurement system.

For continuous scanning tests, the vibration phase of different blades cannot be directly identified. However, that can be derived from the frequency response function of a series of points on all blades. That is to say, to ensure the same installation and excitation conditions as the above continuous scanning test, a series of points is selected at the same position of each blade to obtain the frequency response functions (FRFs). The vibration phase angle of all blades can be determined by the phase frequency curve of FRFs. But for the conditions of unknown excitation, it is impossible to directly measure the FRF, so the reference response is necessary to obtain the ODS FRF of the series of points. The ODS FRFs of each measurement point are measured by calculating the response of the test point with the response of a fixed reference point, from which the relative vibration phase between different blades can be extracted. The selection of measuring points and the fixed reference point used for blade identification of the vibration phase are shown in Figure 7a. The measurement system employed in this experiment is composed of a Polytec-PSV-400 scanning laser vibrometer and a self-developed external scanning system, as presented in Figure 7b. The self-developed external scanning system is used to obtain the response of the measuring points on each blade, while the Polytec-PSV-400 is used to measure the response data of the fixed reference point simultaneously.

#### 3.3.1. Identification of Vibration Phase Based on FRF

In the test of identification of the vibration phase, the electromagnet exciter is also supplied to the sine signal with a frequency of 36.88 Hz, which is exactly the same as the continuous scanning test. A total of 16 measuring points are selected from the same position on each blade and the fixed reference point is located on the tip of the No. 1 blade. The motion equation of the excited structure is given by:

(11) $$M\ddot{x}(t)+C\dot{x}+Kx(t)=F(t)$$

where ***M***, ***C*** and ***K*** are the Mass matrix, damping matrix and stiffness matrix, respectively. F(t) is the vector of excitation force. The frequency response function can be directly derived from the motion equation, and is expressed as:

(12) $$H(\omega )={\left(K-\omega M+i\omega C\right)}^{-1}=\frac{\left\{X\right\}}{\left\{F\right\}}$$

**Figure 8:**
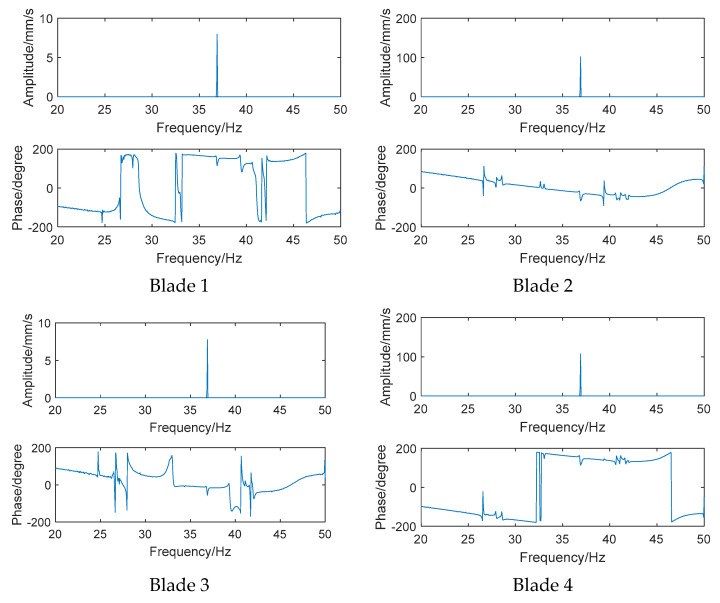

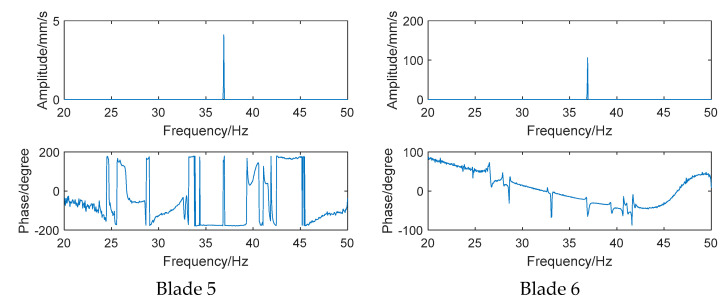
Frequency response functions.

In this paper, the non-contact electromagnet exciter is used to excite the bladed disk and the frequency response function is obtained by dividing the vibration response of the input signal of the exciter [22]. Taking the frequency response function of the previous six measuring points as an example, the frequency response functions of measuring points are shown in Figure 8. It can be found that only one peak appears in the frequency amplitude curve near the excitation frequency of 36.88 Hz.

The vibration phase angles corresponding to the peak frequency are extracted from the phase frequency curve of the 16 measurement points, as shown in Table 3. It is obvious that the difference in phase angle between adjacent the even-numbered blades is about 180°.

#### 3.3.2. Identification of Vibration Phase Based on ODS FRF

When the excitation signal is unknown, the frequency response function of the measurement point cannot be obtained directly. However, the transfer function between the reference point and the measurement point can be obtained by taking the response of a fixed point as a reference signal as follows:

(13) $$T_{xy}(\omega )=\frac{X_{x}(\omega )}{X_{y}(\omega )}$$

where $X_{x}(\omega )$, $X_{y}(\omega )$ are the frequency spectrums of the measuring points and the reference points, respectively. In order to improve the signal-to-noise ratio in practical engineering test, auto power spectrum and cross power spectrum are usually used to optimize the transfer function as follows:

(14) $${\tilde{T}}_{xy}(\omega )=\frac{X_{x}(\omega )\cdot X_{y}^{*}(\omega )}{X_{y}(\omega )\cdot X_{y}^{*}(\omega )}=\frac{G_{xy}(\omega )}{G_{yy}(\omega )}$$

The concept of ODS FRF on the basis of transfer function is first proposed by Richardson M H [23] in 1997. Similar to the transfer function, ODS FRFs are similarly calculated by the auto spectrum and cross-spectrum between the response of fixed reference point and the responses of the measuring points:

(15) $$\mathrm{ODSFRF}(\omega )=\sqrt{G_{xx}(\omega )}\cdot \frac{G_{xy}(\omega )}{\left|G_{xy}(\omega )\right|}=\left|X_{x}(\omega )\right|\cdot \frac{G_{xy}(\omega )}{\left|G_{xy}(\omega )\right|}=\bar{X_{x}}(\omega )$$

**Figure 9:**
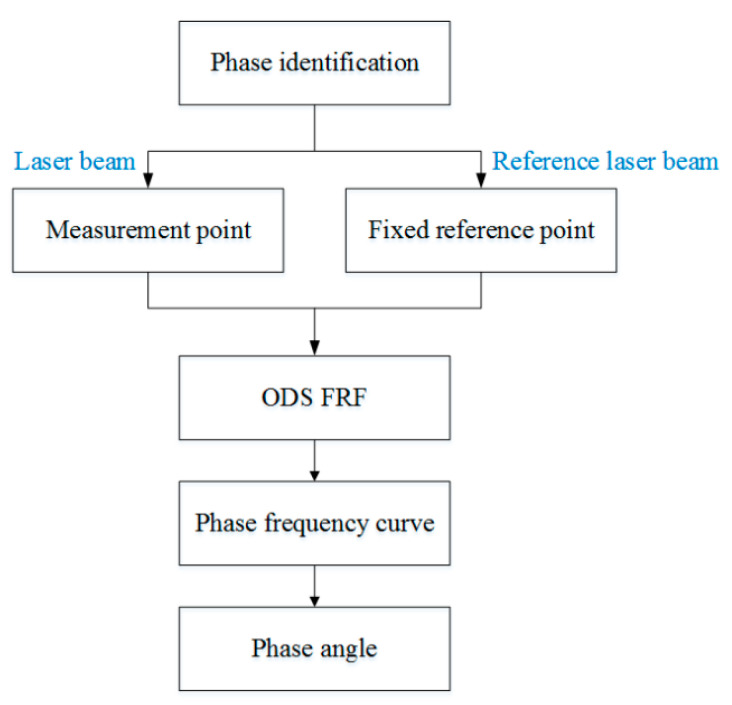
Experiment flow chart.

ODS FRF not only contains the correct amplitude of the response of each measuring point but also the phase information relative to the reference point. Compared with the transfer function, ODS FRF has a higher signal-to-noise ratio [24,25]. According to the ODS FRF theory, the test for vibration phase identification is carried out and makes sure that it is consistent with the condition and environment of the continuous scanning laser test. In the phase identification experiment based on ODS FRF, two laser beams are necessary to obtain the response of the measurement point and the fixed reference point simultaneously, and the experiment flow is shown in Figure 9. 

**Figure 10:**
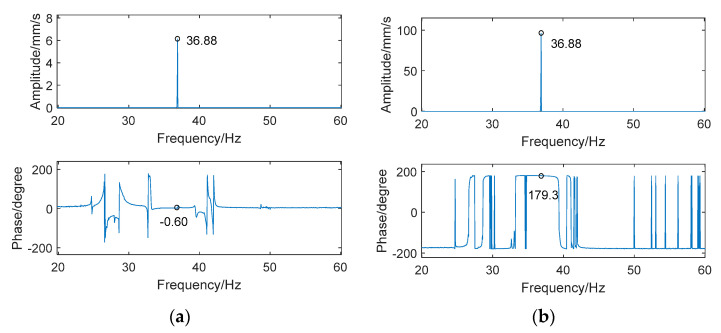
ODS FRF. (**a**) Blade 1. (**b**) Blade 2.

First, a fixed reference response point needs to be selected on the blade disk (see Figure 7). Then, two laser beams are used to simultaneously measure the response of the fixed reference point and the response of each blade measurement point. Take the ODS FRFs measured from blade 1 and blade 2 as an example, the frequency amplitude curves and the phase frequency curves are shown in Figure 10. The phase angles can be easily extracted from the phase frequency curves of ODS FRF. For the two blades, they are −0.6° and 179.3°, respectively.

The phase angles of relative vibration between different blades are extracted from ODS FRFs of measuring points, as shown in Table 4. The results show that the difference in the vibration phase of two adjacent even-numbered blades is about 180°, which signifies that the vibration direction of two adjacent even-numbered blades is opposite. This is consistent with the phase identification results derived from the method based on FRF.

#### 3.3.3. ODS Reconstruction of the Whole-Bladed Disk

**Figure 11:**
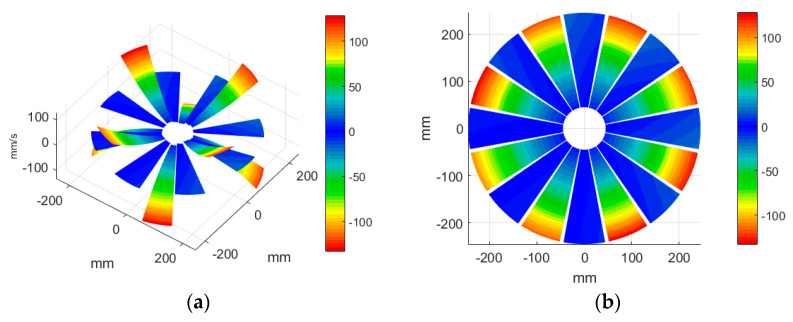
ODS of the whole-bladed disk. (**a**) Three-dimensional perspective. (**b**) Plane perspective.

Combined with the vibration phase angles of all blades identified by the above methods and the ODS of all blades tested in Section 3.2.2, the ODS of the whole-bladed disk is reconstructed, as shown in Figure 11. To verify the accuracy of the measurement, a comparison with the FEM (as presented in Section 3.1) is made. The modal assurance criterion (MAC) value is typically to quantitatively describe the similarity between two modes. The closer the MAC value approaches to 1, the higher the consistency between the two modes. The results showed that the MAC value between the ODS of the bladed disk obtained through testing and the ODS calculated by the finite element method is 0.91. The errors are mainly caused by the fact that the boundary conditions in FEM are not exactly the same as the actual situation, as well as experimental errors, etc.

**Figure 12:**
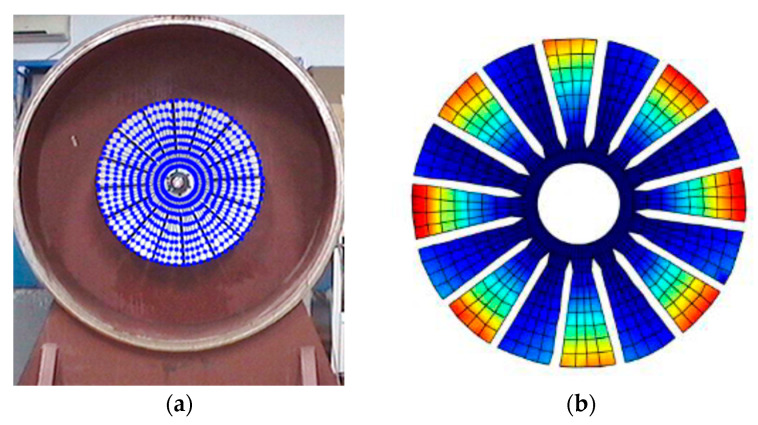
Measurement of ODS in SLDV. (**a**) Arrangement of measuring points. (**b**) The 4-nodal diameter bending mode.

To better investigate the validity and accuracy of the ODS reconstruction method based on ODSFRF in CSLDV, the conventional SLDV method is also employed to conduct experiments in the same installation conditions and experimental environment for comparison. Regarding the test, the fewer the measuring points are, the rougher the obtained modal vibration mode will be. For the test, 5 × 9 measurement points were arranged on each blade, and there are a total of 1440 measurement points on the entire bladed disk as shown in Figure 12a. In order to reduce the random error in the testing process, each measuring point is tested three times, and the single testing time is 4.096 s. The frequency response function adopts the average of three times. It takes about 3 h to complete the entire test. Finally, the four-nodal diameter bending mode of the bladed disk from the test data in SLDV is shown in Figure 12b.

Compared to SLDV, the ODS reconstruction method based on CSLDV only takes a few minutes in terms of time consumption. However, the MAC value between the ODS obtained by the two is as high as 0.97. This demonstrates that the measurement method proposed in this paper is feasible and accurate.

## 4. Conclusions

In this paper, a fast measurement method of operational deflection shapes of bladed disks with CSLDV is presented, in which all blades are tested in the same condition by switching the initial phase of the laser point and a phase identification method based on ODSFRF is proposed to reconstruct the ODS of the whole-bladed disk. In order to determine the relative vibration phase between each blade, two laser beams are needed to participate in the experiment. In this paper, the vibration phases of each blade of the bladed disk are, respectively, identified through the utilization of FRFs and ODSFRFs, and the two methods yield consistent results. However, the ODSFRF is applicable in the situation of unknown excitation. Bladed disks typically rotate at high speeds, and the excitation under operating conditions is rather difficult to measure. Hence, this ODSFRF method is anticipated to be potentially applied in the ODS measurement of rotating bladed disks. According to the ODSs and the relative vibration phase angles of all blades, the ODS of the whole-bladed disk has been reconstructed. The effects of the extended CSLDV measurement method and phase identification of the bladed disk have been investigated experimentally. To validate the accuracy of the ODS reconstructed based on CSLDV and ODSFRF, a discrete point testing experiment in SLDV was carried out supplementarily. The results show that the CSLDV and phase identification method for the bladed disks are feasible and they take less time to obtain the same high-precision ODS. 

## Figures and Tables

**Table 1:** Frequencies of the 1st family of modes.

Order	Frequency/Hz	Nd	Order	Frequency/Hz	Nd
1	26.74	1	9	38.40	4
2	26.74	1	10	41.16	5
3	27.10	0	11	41.16	5
4	28.51	2	12	42.76	6
5	28.51	2	13	42.76	6
6	33.89	3	14	43.61	7
7	33.89	3	15	43.61	7
8	38.40	4	16	43.87	8

**Table 2:** ODSs of all blades.

No.	ODS	No.	ODS
1	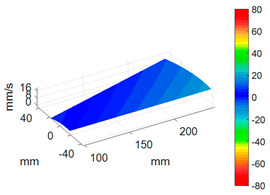	2	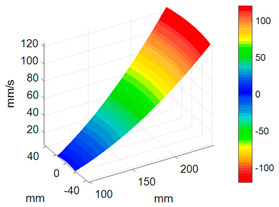
3	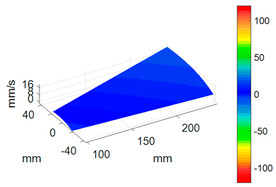	4	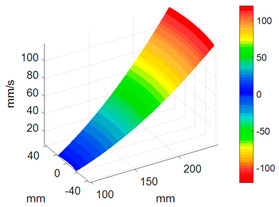
5	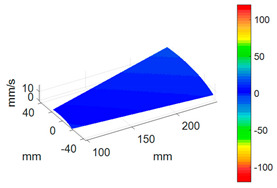	6	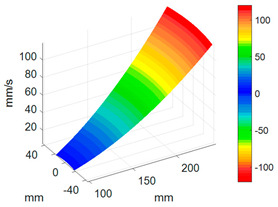
7	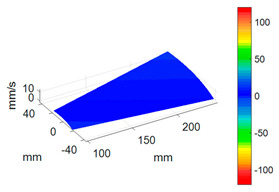	8	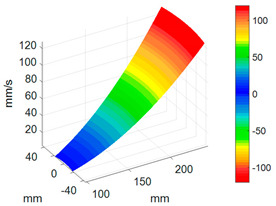
9	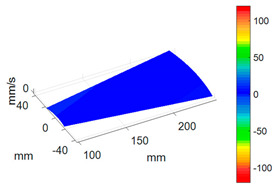	10	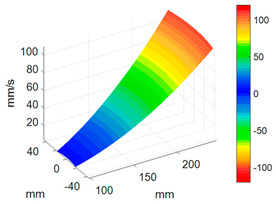
11	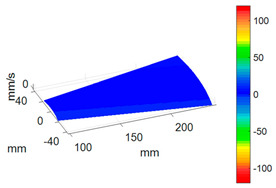	12	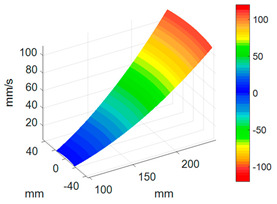
13	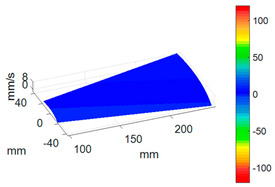	14	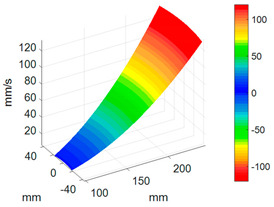
15	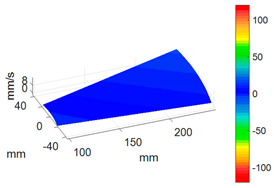	16	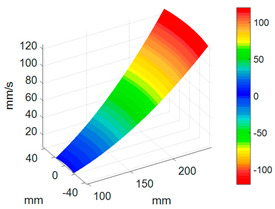

**Table 3:** Phase angles of all blades based on FRF.

No.	Phase Angle/°	No.	Phase Angle/°
1	113.63	2	−67.65
3	−56.86	4	112.8
5	149.26	6	−65.49
7	20.83	8	115.42
9	−4.97	10	−65.03
11	152.42	12	114.58
13	−4.96	14	−64.41
15	−85.40	16	114.76

**Table 4:** Vibration phase angles of all blades based on ODS FRF.

No. of Blade	Phase Angle	No. of Blade	Phase Angle
1	−0.60	2	179.28
3	−171.02	4	−0.79
5	36.01	6	179.24
7	−93.00	8	−0.08
9	−123.11	10	179.5
11	35.16	12	−0.71
13	−123.26	14	179.41
15	156.99	16	−0.66

## Data Availability

Data are contained within the article.

## References

[1] Chatterjee A., Kotambkar M.S. (2015). Modal characteristics of turbine blade packets under lacing wire damage induced mistuning. J. Sound Vib..

[2] Lu Y.H., Mei Q., Lei M.Z., Yu J.J. (2014). Vibration stress test and dynamical evaluation for high rotational speed compressor. J. Propuls. Technol..

[3] Philippe J., Thouverez F., Blanc L., Gruin M. (2018). Vibratory behavior prediction of mistuned stator vane cluster: An industrial application. Comput. Struct..

[4] Tang W., Epureanu B.I., Fillppi S. (2017). Models for blisks with large blends and small mistuning. Mech. Syst. Signal Process..

[5] Lim H.S., Chung J., Yoo H.H. (2009). Modal analysis of a rotating multi-packet blade system. J. Sound Vib..

[6] Witek L., Stachowicz F. (2016). Modal analysis of the turbine blade at complex thermomechanical loads. Strength Mater..

[7] Zhao Z.B., He E.M., Wang H.J. (2011). Experimental Investigation on Natural Characteristics and Forced Response of Bladed Disks. Appl. Mech. Mater..

[8] Yao J., Wang J., Li Q. (2011). Improved Modal Localization and Excitation Factors for Understanding Mistuned Bladed Disk Response. J. Propuls. Power.

[9] Liao H., Wang J., Wang S., Li Q. (2011). Experimental investigation of the worst-case mode localization for a mistuned bladed disk assemblies. J. Aerosp. Power.

[10] Kumar R.R., Pandey K.M. (2017). Static structural and modal analysis of gas Turbine blade. IOP Conf. Ser. Mater. Sci. Eng..

[11] Hancox J., Staples B.C., Parker R.J. (2016). The application of scanning laser Doppler vibrometry in aero-engine development. Proc. Inst. Mech. Eng. Part G J. Aerosp. Eng..

[12] Junge B. (1994). Experiences with scanning laser vibrometry in automotive industries. International Conference on Vibration Measurements by Laser Techniques: Advances and Applications. Int. Soc. Opt. Photonics.

[13] Heinemann T., Becker S. (2017). Axial fan blade vibration assessment under inlet cross-flow conditions using laser scanning vibrometry. Appl. Sci..

[14] Hu Y., Kang Y., Yu K., Zhu W., Zhao R. (2023). Complete operating deflection shapes and model updating for an excited structure in thermal environments via an optimized continuously scanning laser Doppler vibrometer with a two-dimension scan scheme. J. Sound Vib..

[15] Sriram P., Craig J.I., Hanagud S. (1990). A scanning laser Doppler vibrometer for modal testing. Inst. Math. Its Appl..

[16] Halkon B.J., Frizzel S.R., Rothberg S.J. (2003). Vibration measurements using continuous scanning laser vibrometry: Velocity sensitivity model experimental validation. Meas. Sci. Technol..

[17] Stanbridge A.B., Martarelli M., Ewins D.J. (2004). Measuring area vibration mode shapes with a continuous-scan LDV. Measurement.

[18] Stanbridge A.B., Ewins D.J. (1999). Modal testing using a scanning laser Doppler vibrometer. Mech. Syst. Signal Process..

[19] Yuan K., Zhu W.D. (2023). Identification of modal parameters of a model turbine blade with a curved surface under random excitation with a three-dimensional continuously scanning laser Doppler vibrometer system. Measurement.

[20] Yuan K., Zhu W.D. (2022). A novel general-purpose three-dimensional continuously scanning laser Doppler vibrometer system for full-field vibration measurement of a structure with a curved surface. J. Sound Vib..

[21] Boudreau A., Paillard B., Rowntree P. (2002). Broadband vibration measurements using a continuously scanning laser vibrometer. Meas. Sci. Technol..

[22] Liu C., Zang C., Zhou B. (2020). Extension of continuous scanning laser Doppler vibrometry measurement for complex structures with curved surfaces. Chin. J. Aeronaut..

[23] Liu C.H., Zang C.P., Li F., Petrov E.P. (2020). High Frequency Modal Testing of the Multiblade Packets Using a Noncontact Measurement and Excitation System. Shock Vib..

[24] Richardson M.H. (1997). Is it a mode shape, or an operating deflection shape?. Sound Vib..

[25] Zhang Y.L., Zhang L.M., Sun W.G., Qiu F.L. (2014). Modal contribution analysis of intercity rails under working conditions based on ODS FRF. Noise Vib. Control.

